# Case Report: Depression × dementia with Lewy bodies in the elderly: The importance of differential diagnosis

**DOI:** 10.3389/fpsyt.2022.1059150

**Published:** 2022-12-19

**Authors:** Alexandre M. Valença, Cláudia Cristina Studart Leal, Gustavo C. Oliveira, Talvane M. de Moraes, Antonio E. Nardi, Mauro V. Mendlowicz

**Affiliations:** ^1^Department of Psychiatry, Federal University of Rio de Janeiro, Rio de Janeiro, Brazil; ^2^Department of Psychiatry, University Center of Brasilia, Brasília, Brazil; ^3^Department of Psychiatry, University of Brasília, Brasília, Brazil; ^4^Department of Psychiatry, Academia Nacional de Medicina, Rio de Janeiro, Brazil; ^5^Department of Psychiatry, Fluminense Federal University, Rio de Janeiro, Brazil

**Keywords:** depression, dementia, Lewy bodies (LBD), diagnosis, legal medicine assessment

## Abstract

**Background:**

Dementia is a clinical syndrome which is more common in elderly people. Dementia with Lewy bodies (LBD) is not so rare in elderly people, with cognitive impairment in about 30% over age 65. The clinical picture is characterized by fluctuation in cognitive functions, recurrent, well-formed, detailed visual hallucinations, and Parkinsonism, with rigidity, tremor, bradykinesia, and slurred speech.

**Case presentation:**

We present a case report of LBD in a 73-year-old retired teacher, which a initial wrong diagnosis of refractory depression for at least 3 years. We also conduct a review of recent works on theme.

**Conclusion:**

LBD diagnosis can be neglected for years, with a legal and clinical issues to patients and their families. Detailed medical research, including differential diagnosis, are very necessary on those cases, specially when they are called refractory. We encourage new research and adequate clinical training to prevent damage.

## Introduction

Dementia is a clinical syndrome characterized by multiple, acquired, and persistent cognitive deficits, capable of substantially interfering in the patient’s daily activities. It is more prevalent in segments of the population with advanced age, especially in those older than 75 years. The increase in the population with dementia is a major concern for health professionals and lawmakers around the world (1). Alzheimer’s disease (AD) and dementia with Lewy bodies (LBD) are the main representative types of the neurodegenerative dementias (2).

LBD is a common form of cognitive impairment, accounting for 30% of cases of dementia in people over age 65. Early diagnosis of LBD can be challenging, particularly in the context of the differentiation between Parkinson’s disease dementia and other forms of dementia, such as Alzheimer’s disease, and mood disorders such as depression (3).

The clinical picture of LBD is characterized by fluctuation in cognitive functions, recurrent, well-formed, detailed visual hallucinations, and Parkinsonism, with rigidity, tremor, bradykinesia, and slurred speech. Memory deficit usually occurs later, and attention deficits, cognitive impairment, and loss of visual-spatial skills become more frequent. Other characteristics are behavioral REM (rapid eye movement) sleep disorder, increased sensitivity to the adverse effects of antipsychotics, and reduced dopamine uptake in the basal ganglia (4).

A retrospective study found that the diagnosis of major depression was initially made in 19% of the 962 patients with LBD (5). Seventeen of the 90 patients with probable LBD (18.9%) reported depression and concomitant antidepressant use before or at the onset of memory loss. The mean prodromal duration of depression before the onset of memory loss was 7.2 ± 12.0 years (6).

Depression in elderly people, diagnosed as senile depression (SD), includes heterogeneous symptoms and clinical profile findings. The pathophysiology remains unclear because it should be different (7). SD may be risk factor for developing dementia or a prodromal stage. Disturbance of neural circuity, imbalance of monoaminergic systems, dysregulation of the hypothalamic–pituitary–adrenal axis, and elevated neuroinflammatory status where studied and are involved with the syndrome (7). A very recent Guideline from Japanese Society of Mood disorders (8) (2022) of diagnosis and treatment of SD emphasizes the need of differential diagnosis from bipolar disorders, organic brain diseases, drug effects and dementia. Determine the comorbidity between late-life depression and dementia is also necessary, according to this society (8).

The objective of this report is to describe and analyze a case of LBD that for several years was erroneously diagnosed and treated as depression.

## Case description

Mr. John is a 73-year-old retired teacher. His wife had requested a Social Security disability benefit for him, but the psychiatric examiner made a diagnosis of “major depression” and the benefit was denied. According to the patient’s wife, he began complaining about “forgetfulness” early in 2015. She noticed “strange things,” such as “sleep attacks,” even in front of the guests invited by the couple. The “forgetfulness” remained relatively mild for three years. However, a further decline in Mr. John’s cognitive skills was observed from 2018 on, when, for instance, he went shopping in a grocery store and forgot several items there but could not acknowledge that loss.

In 2020, the memory deficits of Mr. John became more severe. On one occasion, he attempted to drive his car but could not find the accelerator pedal. This same year, he got lost trying to get to his son’s home and had to ask for directions, although the neighborhood was quite familiar to him. Mr. John could not understand movies or TV shows anymore. He complained of sadness, anguish, ideas of death, and of not sleeping all night. Several psychiatrists assessed the patient during the last five years, made a diagnosis of refractory depression, and medicated him with a variety antidepressant drugs. At the beginning of 2021, Mr. John underwent electroconvulsive therapy that resulted in limited improvement.

The patient’s depression was accompanied by cognitive problems, such as forgetting events, appointments, and people’s names and frequent losses of his belongings. He reported false memories, such as having been present at a specific musical show, a fact that never really did occur. Mr. John also reported visions of “dead people and butterflies.”

## Diagnostic

There were report of episodes of exacerbation of the parkinsonism with the prescription of antipsychotic medications such as quetiapine. After being medicated with this drug, the patient remained in bed for three days due to severe muscle rigidity. He walked very slowly, took a long time to eat meals, and had hand tremors. He suffered several falls to the ground while standing or walking. His wife reported that he had many nights of agitated sleep, in which he talked almost non-stop, and repeatedly moved his arms.

A neuropsychological assessment carried out in 2021 identified difficulties in recognizing shapes and parts of objects and of integrating them into a whole. The patient also showed deficits in attention and cognitive flexibility, in the ability to plan and monitor tasks, in the inhibitory control, and in verbal fluency. In a screening test, he could spontaneous say 9 animals’ names in one minute. According to Brucki et al. (9), 13 names is expected in Brazilian population. In 2019, it was related that the patient could say 20 names doing the same test, for comparison. Now, in this neuropsychological it was also found: five digit test (FDT) showed less than 5 score in 95,9“ and 74,8” for alternance and flexibility respectively, which is a severe impairment. WAIS-III (Wechsler Adult Intelligence Scale) showed operational memory affected. Other findings included impairment in the ability of naming figures, to express the meaning of words, and of learning new material, after previous exposure to new content.

**FIGURE 1:**
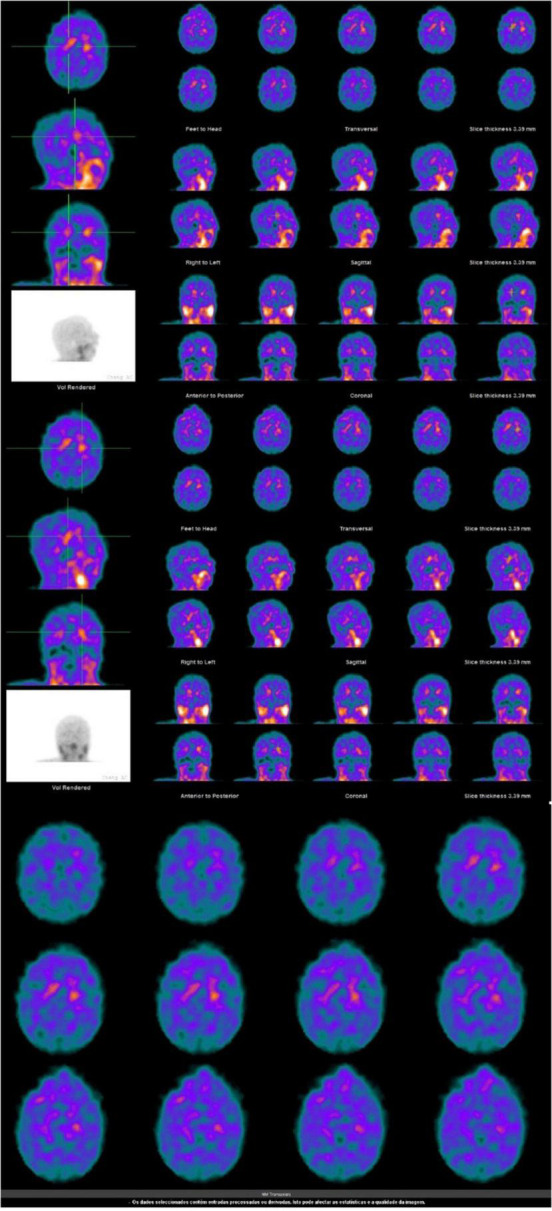
Patient’s exams.

Cerebrospinal fluid analysis of neurodegeneration biomarkers identified increased levels of T-Tau protein and decreased levels of beta-amyloid ratio (AB42/AB40). Nuclear magnetic resonance imaging (MRI) of the skull showed foci of ischemic gliosis at the posterior margins of the ventricular bodies. Preservation of neurons in the pars substantia nigra and hippocampus. Dilated supratentorial ventricles, with type 4 hydrocephalus described. Spectral analyses of MRI with proton spectroscopy revealed glutamine/glutamate peaks and increased levels of myo-inositol in the frontal lobes, notably in the right one. Basal cisterns, fissures, and cerebral convexity grooves were more evident, notably in high parietal convexities bilaterally. Scintigraphy of the dopaminergic neurons of the base nuclei showed a bilaterally reduced concentration of the radiotracer in the basal nuclei, a finding compatible with severe nigrostriatal dopaminergic dysfunction (Figure 1). Despite the increase of T-tau protein usually indicates the presence of Alzheimer Disease, the decrease of AB42/AB40 is not going to confirm. The alterations in image exams and the detailed clinical history of our case are more suggestive to LBD (10).

Dopamine transporter (DAT) single-photon emission tomography showed a markedly reduced bilateral concentration of the radiotracer in the basal ganglia, a finding that is compatible with severe nigrostriatal dopaminergic depletion. The usefulness of this test in distinguishing dementia with LBD from Alzheimer’s disease is well established, having a sensitivity of 88% and specificity of 100% in the exclusion of cases with other dementias other than LDB (3).

**FIGURE 2:**
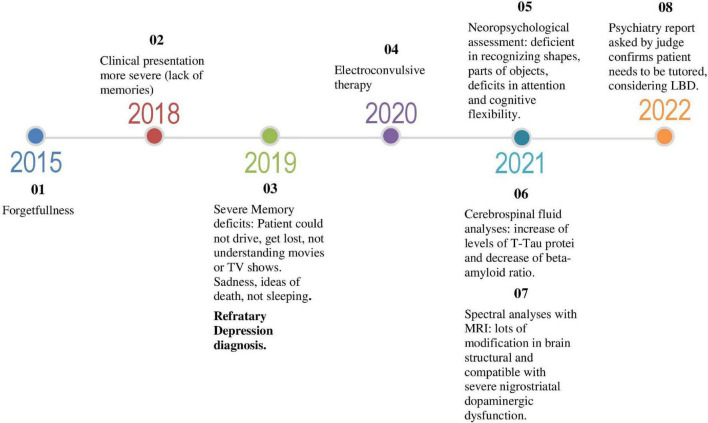
Time-line.

The latest mental state examination was performed three months ago. The patient was awake and fully oriented in time and space. However, he was unable to remember several relevant information, like the year he retired or his wedding day. Mr. John was little attentive to the interview, and it was necessary to repeat questions or rephrase them to further his understanding. Data about his psychiatric history had to be provided by his wife, and he was unable to establish a chronological link between his complaints and the symptoms he presented. Memories of recent and remote events were impaired. There are reports of paramnesias (memories of events that do not correspond to reality). The Mini-mental state examination (MMSE) was applied and the score was 25/30. Orientation and recent memory were affected with loss of 1 point of score each, and loss of attention was the responsible for other 3 points loss. The decrease of verbal fluency was confirmed with just 9 names spontaneously spoken by him. Mr. John complained about “depression, discouragement, forgetfulness, and bad mind.” The content of his thinking was poor, and the flow of ideas was impaired. He reported visual hallucinations in the past. His mood was apathetic and the affectivity was faded. Willpower and pragmatism were greatly impaired. His wife said he still affected, even using Rivastigmin for near to one year. He is also using Olanzapine, Mirtazapine, Zolpidem, Pramipexole and Alprazolam. She considers that he is calmer, but with some changes in his mood, which are not very predictable. He is probably stable, not better or worse than a year ago in cognition skills, and he still need support in his self-care. On the other hand, his behavior and his sleep are better now. The time-line (Figure 2) show the case progression.

## Discussion

Holistically, the examination of the patient’s mental state, added by ancillary exams, neuropsychological evaluation, and history data, strongly points to the presence of degenerative dementias, such as Alzheimer’s dementia and LBD (11, 12).

According to the Alzheimer’s Association (13), LBD can occur either alone or in association with Alzheimer’s disease or with vascular dementia. There are many studies in Alzheimer’s disease about its neuropathological perspective Lewy Bodies, plaques, tangles and proteins may increase the risk of Dementia, in different perspective. LBD seems clearly to affect younger ages than the usual modification from Alzheimer’s and other Dementia (14). Mr. John also had several signs and symptoms related to LBD, such as visual hallucinations, hypersensitivity to antipsychotics, neuropsychiatric symptoms, parkinsonism, and behavioral sleep disorder. Regarding visual hallucinations in LBD they are complex in terms of content and can be well-structured, vivid, and detailed, involving people or animals. More than 80% of patients with this type of dementia report these experiences (15), as in the case in question. In these cases, there is also a hypersensitivity to antipsychotic drugs. Even the hypersensitivity to antipsychotics has become a support criterion for the diagnosis of LBD (16). In the present case, Mr. John presented severe extrapyramidal symptoms after the use of quetiapine and it continued despite stopping this medication.

The patient also had depression and reported previous suicidal ideation. This is a common neuropsychiatric symptom in LBD. One cannot fail to consider the impact of dementia on the current depressive condition, including contributing to the genesis of depression, its worsened prognosis, and its refractoriness to pharmacological treatment and electroconvulsive therapy, as in the present case.

The patient has presented symptoms of parkinsonism in his daily life, such as postural instability (report of falls), bradykinesia, and slowing of movements. It is important to note that spontaneous parkinsonism affects more than 85% of patients with LBD (17).

It is important to note that although LBD was not listed in ICD-10 (18) but in ICD 11 (19) it is featured as a dementia associated with Lewy body disease (6D82). ICD-11 (19) describes it as follows: “Dementia associated with Lewy body disease is the second most common form of dementia in elderly disease after Alzheimer’s.” The onset is insidious, with attention and executive functioning deficits usually reported as the complaint initially presented. These cognitive deficits are often accompanied by visual hallucinations and symptoms of REM sleep behavioral disorder. Hallucinations in other sensory modalities, depressive symptoms, and delusions may also be present. The presentation of symptoms generally varies significantly over days requiring longitudinal observation. The spontaneous onset of parkinsonism within approximately 1 year of the onset of cognitive symptoms is characteristic of the disease.” It should be noted that this description corresponds entirely to the case in question. According to the last Consensus of LBD (2017) (20) new information has been incorporated about aspects of DLB, with increased diagnostic weighting given to REM sleep behavior disorder and 123iodine-metaiodobenzylguanidine (MIBG) myocardial scintigraphy. The patient hasn’t do does tests, but his sleep became bad, since the diagnosis and the disease progress. Despite that, the case can be consider a probable LBD, as he has Fluctuating cognition with pronounced variations in attention, recurrent visual hallucinations well-formed and detailed and rigidity, rest tremor, rigidity and bradykinesia, with at least 3 clinical features, so not just the positive biomarkers. The treatment is also based on specialist opinion, as there are few randomized controlled trials in DLB, also according to consensus (20).

## Patient perspective

A case as the reported brings many problems to the patient and his family, as a late diagnosis implied inadequate therapy, with side effects resulting from this, as reported. In addition, legal repercussions are a big issue, as a diagnosis of refractory depression rather than dementia, which is a serious mental illness. This situation limits patient’s access to rights and even to a better care, through tutoring, for example, and it also become difficult to their families.

## Conclusion

The present case illustrates how in a dementia syndrome related to LBD, cognitive functioning and the social and daily life of the individual are greatly impaired. The study of clinical, neurological, and psychopathological characteristics of patients with depression who present symptoms of degenerative dementias such as LBD can contribute to elucidating the differential diagnosis between these conditions. Depression in old age can be a prodromic presentation of degenerative dementias (15). Certainly, the study of this relationship deserves to be further studied.

Detailed medical research is extremely important in these cases, including differential diagnosis with depression, to provide adequate treatment, and family guidance and enable financial benefits provided by social security when necessary, thus contributing to social justice and better quality of life for these patients.

## Data availability statement

The original contributions presented in this study are included in the article/supplementary material, further inquiries can be directed to the corresponding author.

## Ethics statement

Ethical review and approval was not required for the study on human participants in accordance with the local legislation and institutional requirements. The patients/participants provided their written informed consent to participate in this study. The patient’s next of kin/legal guardian provided written informed consent for the publication of this case report.

## Author contributions

AN, TM, GO, and CS contributed to conception and design of the study, analyzed the case, and reviewed literature. MM reviewed and organized the sections of the manuscript, rewriting parts of the manuscript. AV wrote the first draft of the manuscript and support review of publication. All authors contributed to manuscript revision, read, and approved the submitted version.
